# Network Pharmacology Analysis of Traditional Chinese Medicine Formula Shuang Di Shou Zhen Tablets Treating Nonexudative Age-Related Macular Degeneration

**DOI:** 10.1155/2021/6657521

**Published:** 2021-03-24

**Authors:** Yue Fang, Xinquan Liu, Jing Su

**Affiliations:** Department of Ophthalmology, Longhua Hospital Affiliated Shanghai University of Traditional Chinese Medicine, Shanghai 200032, China

## Abstract

**Objective:**

To analyze the pharmacological mechanism of the treatment of dry age-related macular degeneration (dry AMD) based on a network pharmacological approach of Shuang Di Shou Zhen Tablets (SDSZT) and to provide a new reference for the current lack of effective treatment of dry AMD.

**Methods:**

The main chemical constituents and their targets of *Rehmanniae Radix Praeparata, Ligustrum lucidum, Mori Fructus, Paeonia albiflora, Rhizoma Dioscoreae, Alisma orientale, Schisandra chinensis, Radix Polygoni Multiflori Preparata, Ophiopogon japonicus,* and *Radix Rehmanniae* were obtained from the Traditional Chinese Medicine Systems Pharmacology Database and Analysis Platform (TCMSP) and Traditional Chinese Medicine Integrated Database (TCMID). The active ingredients of traditional Chinese medicine were screened according to Absorption, Distribution, Metabolism, and Excretion (ADME), the gene names of the targets of each active ingredient were obtained from the Uniprot database, the main targets of dry AMD were obtained from GeneCards and DisGeNET database, and the protein interaction analysis was performed on the String database. The Metascape database was used to analyze the “drug-component-target” and the biological processes and networks involved, and then, Cytoscape 3.8.1 was used to construct the “ SDSZT component-dry AMD target-pathway” network.

**Results:**

The main active ingredients of SDSZT for dry AMD treatment are quercetin, kaempferol, luteolin, *β*-glutamine, *β*-carotene, etc. And, the core targets are RAC-alpha serine/threonine-protein kinase (AKT1), prostaglandin G/H synthase 1 (PTGS1), tumor necrosis factor (TNF), transcription factor AP-1 (JUN), apoptosis regulator Bcl-2 (BCL2), caspase-3 (CASP3), phosphatidylinositol 4,5-bisphosphate 3-kinase catalytic subunit gamma isoform (PIK3CG), androgen receptor (AR), apoptosis regulator BAX (BAX), etc. The biological pathways for the treatment of age-related macular degeneration by SDSZT mainly act on pathways in cancer, fluid shear stress and atherosclerosis, and TNF signaling pathway, and the main function of SDSZT is to regulate intracellular cytokine receptor binding.

**Conclusion:**

This study initially reveals the multiconstituent, multitarget, and multipathway mechanism of action of SDSZT in the treatment of dry AMD and provides the basis for the clinical application of SDSZT.

## 1. Introduction

Age-related macular degeneration (AMD) is one of the most common eye diseases in ophthalmology and is one of the leading causes of blindness in the elderly population [1]. It can be divided into nonexudative AMD (dry AMD) and neovascular AMD (wet AMD). Existing anti-vascular endothelial growth factor (anti-VEGF) is known to be effective in the treatment of wet AMD; however, there is a lack of definitive and effective therapeutic measures for dry AMD. In recent years, there have been numerous studies on the treatment of dry AMD, such as complement factors [2], anti-inflammatory therapy [3], acupuncture [4], and traditional Chinese medicine [5, 6]. However, none of these can be considered definitive and effective treatments.

The advantages of traditional medicine in treating AMD should not be overlooked. The treatment mechanism of traditional Chinese medicine is not only to a single target, but is also based on the holistic view of traditional Chinese medicine to provide a multitarget, multifaceted, and comprehensive treatment of the disease, with high safety. Network pharmacology is the use of known drug components, component targets, and known targets of disease to predict the likelihood of a drug's potential to treat a disease, and the network is able to comprehensively respond to possible mechanisms of drug interventions. Through multipathway modulation of signaling pathways, network pharmacology, as a new tool for drug research, can effectively tap into the active ingredients of traditional Chinese medicine, discard toxic ingredients, and treat the corresponding diseases with maximum stability and precision.

Shuang Di Shou Zhen Tablet (SDSZT) is a self-made ophthalmic medicine for Longhua Hospital, Shanghai. For many years, SDSZTs have been used in ophthalmology clinics to treat age-related eye diseases (deficiency of liver-yin and kidney-yin type: The deficiency of liver and kidney in traditional Chinese medicine, which means patients with dim eyesight, metamorphosis, drusen, and pigmentary disturbance in macula, accompanied by general symptoms of dizziness, tinnitus, lassitude in loin and legs, blackish tongue with thin coating, and thready thin pulse). In preliminary clinical observations, it was found that treatment with vitamin C and vitamin E combined with SDSZT resulted in a smaller number of Amsler table deformation frames and a smaller extent of central visual field defects in patients with dry AMD compared to vitamin C and vitamin E alone [6, 7]. This formula contains 10 herbs: *Rehmanniae Radix Praeparata, Ligustrum lucidum, Mori Fructus, Paeonia albiflora, Rhizoma Dioscoreae, Alisma orientale, Schisandra chinensis, Radix Polygoni Multiflori Preparata, Ophiopogon japonicus*, and *Radix Rehmanniae,* of which *Rehmanniae Radix Praeparata* and *Ligustrum lucidum* are popular herbs for research on their antioxidant, anti-inflammatory, and lipid metabolizing components in recent years [8–12]. Therefore, this study, based on network pharmacology approach, explores the active ingredient targets of SDSZT and the molecular mechanism of its treatment for dry AMD disease and provides a certain theoretical basis for the follow-up study.

## 2. Materials and Methods

### 2.1. Potential Drug Targets Prediction for SDSZT

The websites of TCMSP (Traditional Chinese Medicine Systems Pharmacology Database and Analysis Platform) (http://lsp.nwu.edu.cn/tcmsp.php) [13] and TCMID (Traditional Chinese Medicine Integrated Database) (http://119.3.41.228:8000/tcmid/) [14] were used to query the chemical composition of 10 TCM herbal ingredients, and ADME values of oral bioavailability (OB) ≥30% and drug-likeness (DL) ≥ 0.18 [15–17] were set for the active ingredients. Preliminary screening was done to obtain active ingredients and targets for each active ingredient. After the screening, the gene names of the relative targets were obtained within the Uniprot protein database (http://www.uniprot.org) for specification. The drug-component-target intersection was mapped by Cytoscape 3.7.1 software [18].

### 2.2. Collection of Known AMD-Related Targets

Using “dry age-related macular degeneration” and “nonexecutive age-related macular degeneration” as keywords, we explored the GeneCards database [19] (http://www.genecards.org) and DisGeNET database [20] (https://www.disgenet.org) for potential targets related to dry AMD. Although higher scores in the GeneCards database indicate a strong association between the target and the disease, there were too few relevant targets for dry AMD in several databases (1056 relevant targets in the GeneCards database and only 12 relevant targets in the DisGeNET database) and therefore no targets greater than the median were set as potential targets here. After merging the targets in the two databases, the potential targets for dry AMD can be obtained by deleting the duplicate targets.

### 2.3. SDSZT-Dry AMD PPI Data

To clarify the interactions between SDSZT-associated targets and dry AMD-related targets, we first used the Venny 2.1.0 (https://bioinfogp.cnb.csic.es/tools/venny/) to get intersection targets. The acquired targets are then used to construct a protein-polymer interaction (PPI) network model from the STRING 11.0 database [21, 22] (http://string-db.org). The biological genus is set to “Homo sapiens” and the minimum required interaction score is set to “highest confidence” (>0.900); the rest are default settings to generate the PPI network diagram. Using Cytoscape 3.7.1, the network is analyzed by CytoNCA to calculate the betweeness centrality (BC), closeness centrality (CC), degree centrality (DC), and the eigenvector centrality (EC); the mean value is calculated and then filtered. The PPI network was further analyzed using the MCODE [23] plugin in Cytoscape 3.7.1.

### 2.4. Gene Ontology and Pathway Enrichment Analysis

The combination of functional enrichment, interactomic analysis, gene annotation, and search in Metascape is an effective tool for comprehensive analysis and interpretation of OMICs-based research in the era of big data [24]. Therefore, SDSZT-dry AMD cross-targets were imported into Metascape (http://metascape.org/gp/index.html), the *P* value < 0.01 was set, and enrichment analysis was performed. Using the Metascape database and the Microbiology Letter Platform (http://www.bioinformatics.com.cn/), we have conducted a number of studies on molecular function (MF), biological process (BP), and cellular component (CC). The top 20 functionally annotated catalogs and signaling pathways are selected for analysis, and the results are presented as bar and bubble plots.

### 2.5. SDSZT-Dry AMD-Pathway PPI Data

Cytoscape 3.7.1 was used to construct a network diagram of the constituents of SDSZT-dry AMD target-pathway network, and Cytoscape 3.7.1 built-in tools were used to analyze the network topology parameters of the active ingredients and targets, including Degree, BetweennessCentrality, and ClosenessCentrality. The network topology parameters were then used to determine the core targets and the main active ingredients that exerted drug effects.

## 3. Results

### 3.1. Candidate Compound Screening for SDSZT and Potential Target Prediction for SDSZT

**Figure 1:**
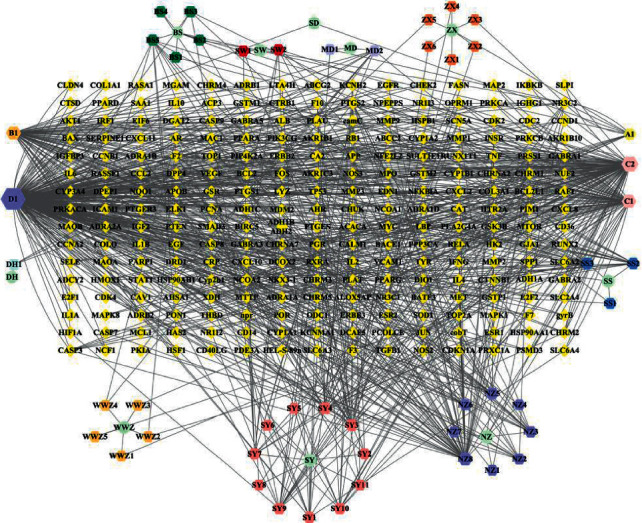
Construction of the SDSZT compound-potential target network. The compound-potential target network was constructed by linking the candidate compounds and their potential targets of the 10 herbs, which are constituents of SDSZT. Squares are for traditional Chinese medicine; hexagons are compound components; abbreviations for compounds can be found in Table 1; diamonds are targets.

Initially, we obtained 327 kinds of chemical compositions of *Rehmanniae Radix Praeparata*, 1042 kinds of *Ligustrum lucidum*, 2190 kinds of *Mori Fructus*, 1007 kinds of *Paeonia albiflora*, 871 kinds of *Rhizoma Dioscoreae*, 179 kinds of *Alisma orientale*, 39 kinds of *Schisandra chinensis*, 22 kinds of *Radix Polygoni Multiflori Preparata*, 60 kinds of *Ophiopogon japonicus*, and 49 kinds of *Radix Rehmanniae.* After ADME screening, there were 2 kinds of chemical composition in *Rehmanniae Radix Praeparata*, 10 kinds of *Ligustrum lucidum*, 6 kinds of *Mori Fructus*, 6 kinds of *Paeonia albiflora*, 15 kinds of *Rhizoma Dioscoreae*, 7 kinds of *Alisma orientale*, 5 kinds of *Schisandra chinensi*, 4 kinds of *Radix Polygoni Multiflori Preparat*, 2 kinds of *Ophiopogon japonicus*, and 2 kinds of *Radix Rehmannia* (Table 1). Finally, the chemical targets were 34 for *Rehmanniae Radix Praeparata*, 360 for *Ligustrum lucidum*, 195 for *Mori Fructus*, 123 for *Paeonia albiflora*, 144 for *Rhizoma Dioscoreae*, 9 for *Alisma orientale*, 7 for *Schisandra chinensia*, 49 for *Radix Polygoni Multiflori Preparat*, 12 for *Ophiopogon japonicu*, and 4 for *Radix Rehmannia*. After merging and deleting duplicate values, a total of 247 drug targets were collated. We then created a network diagram of the SDSZT herbs-active ingredient-drug target (Figure 1).

### 3.2. Collection of Known Dry AMD-Related Targets

1056 dry AMD targets were obtained from the GeneCards database, and due to the small number of targets obtained, the score range was not set for screening. In combination with the DisGeNET database, 11 targets were obtained for nonexudative age-related macular degeneration, and 1059 dry AMD targets were obtained for dry AMD after deleting the duplicates.

### 3.3. SDSZT-Dry AMD PPI Data

**Figure 2:**
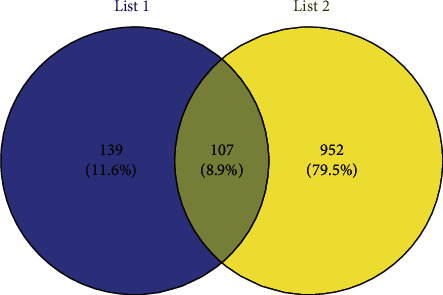
SDSZT component targets (list 1); dry AMD targets (list 2); Venny chart.

**Figure 3:**
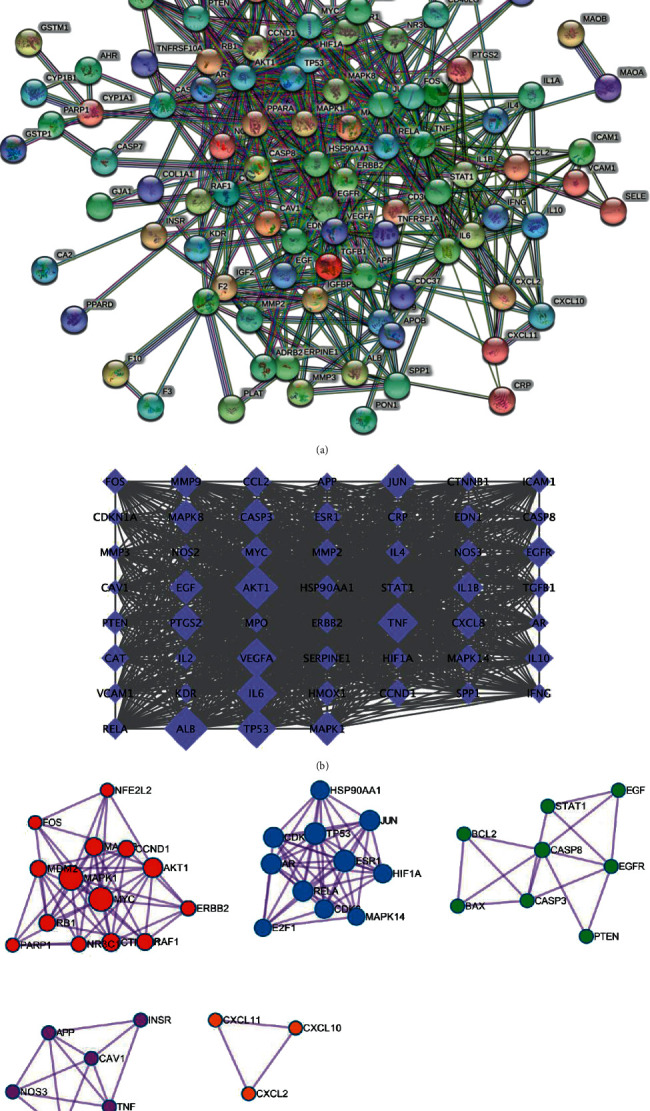
Protein-protein interaction networks. The mapping of PPI network was generated by the STRING server. (a) SDSZT-dry AMD targets PPI network (String): 107 main target genes regulated by SDSZT formula in the treatment of dry AMD and its complications. This network contains 107 nodes and 2210 edges. (b) PPI network of SDSZT-dry AMD key target. As shown in (b), the larger area of the rhombus could be considered as more important in this network. The degree value of each node in Figure 2(c) is not presented in article. (c) Module in a PPI network for SDSZT-dry AMD targets.

The screened targets of SDSZT active ingredients and dry AMD disease were imported into the online Venny analysis website, and the intersection targets were plotted in the Venny plot, resulting in 107 common targets of the ingredients of SDSZT and dry AMD obtained (Figure 2). The obtained intersection targets were imported into the STRING 11.0 platform, and the PPI network of SDSZT was obtained, with 2210 interacting edges and an average node degree value of 41.3 (Figure 3(a)). In order to further clarify the potential targets and protein-protein interactions at key nodes, the CSV files obtained in STRING 11.0 were imported into Cytoscape 3.7.1 software, and the network was analyzed by cytoNCA. The calculated values of BC, CC, DC, and EC were 0.776567196, 0.623529412, 42, and 0.091691375, respectively. Fifty-three core targets were selected based on greater-than-mean values (Figure 3(b)). Among them, ALB, AKT1, IL6, TNF, TP53, VEGFA, CASP3, PTGS2, JUN, and MAPK1 are the top 10 targets of degree value. There are tightly connected regions in the PPI network, which may represent molecular complexes called module [25], and the MCODE plugin in Cytoscape 3.7.1 is used to mine the interacting tightly connected regions in the PPI network to obtain the modules (Figure 3(c)).

### 3.4. Gene Ontology and Pathway Enrichment Analysis

The signaling pathways of the targets associated with SDSZT for dry AMD were obtained from the analysis of the Metascape website. The results were then visualized by Cytoscape 3.7.1 software and the Microbiology platform. The result is a target that is inextricably linked to dry AMD.

**Figure 4:**
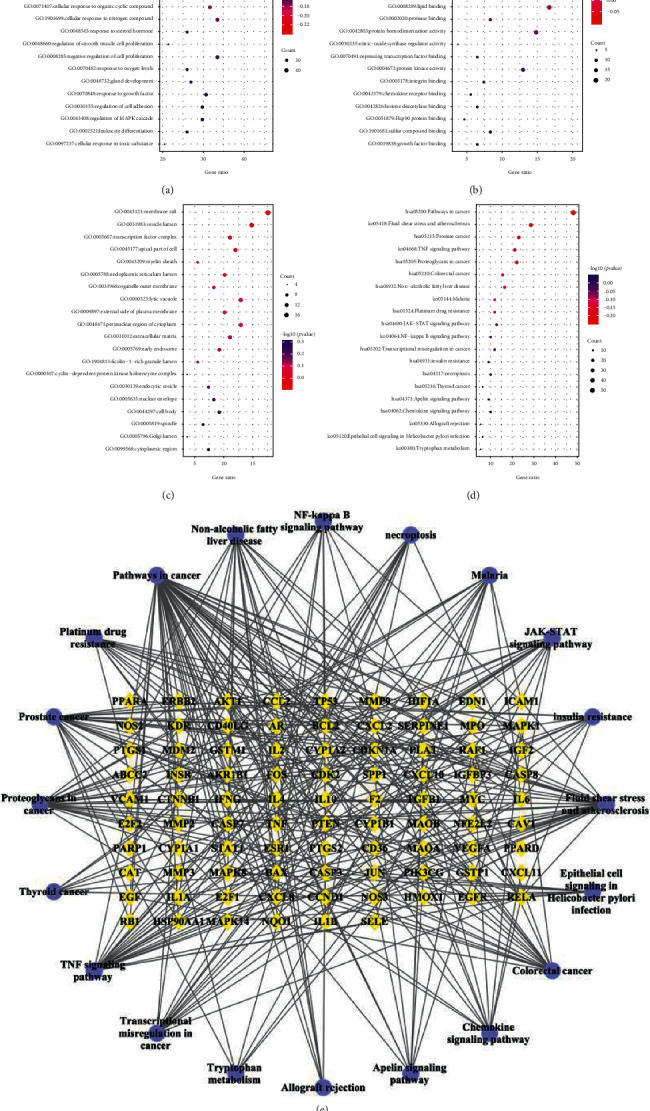
Enrichment analysis of potential targets for the main components of SDSZT. (a): GO-BP analysis; (b): GO-MF analysis; (c): GO-CC analysis; (d): KEGG analysis. (e): Intersection of the top 20 KEGG pathways and targets after screening, with hexagonal pathways and rhombuses as common targets.

The main biological processes involved in SDSZT include response to inorganic substance, response to toxic substance, response to lipopolysaccharide, and reactive oxygen species metabolic process, apoptotic signaling pathway, response to wounding, and response to extracellular stimulus (Figure 4(a)). The main pathways involved are pathways in cancer, fluid shear stress and atherosclerosis, and TNF signaling pathway (Figure 4(d)). The enrichment targets of target-pathway enrichment results are shown in Table 2, and the top 20 filtered pathway-target network diagrams produced by Cytoscape 3.7.1 are shown in Figure 4(e).

Relevant targets regulate the function of dry AMD, which is mainly enriched in cytokine receptor binding, transcription factor binding, protein domain specific binding, kinase binding, phosphatase binding, antioxidant activity, and oxidoreductase activity (Figure 4(b)).

### 3.5. SDSZT-Dry AMD-Pathway PPI Data

**Figure 5:**
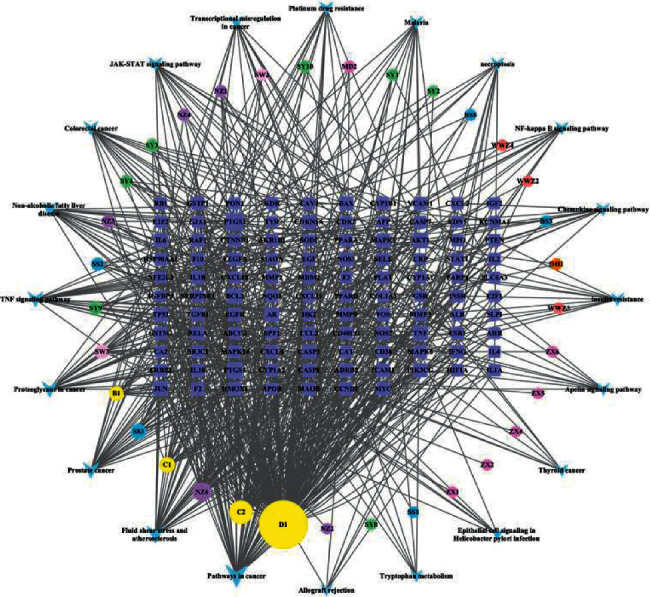
SDSZT component-dry AMD target-pathway network diagram. Triangles are pathways; circles are active components of drugs; different colors are components of different drugs; squares are targets shared by disease drugs. Refer to Table 1 for Chinese herbal abbreviations.

Cytoscape 3.7.1 analyzed the SDSZT-disease target-pathway network, as shown in Figure 5. By backpropagation, 33 compounds of SDSZT acting on dry AMD were obtained, and the network diagram included 725 nodes. The topological parameters of the SDSZT network were analyzed by NetworkAnalyzer, and the core components and core targets were obtained. The analysis showed that D1 quercetin had a degree of 162, a betweenness of 0.4159, and a closeness of 0.608, predicting quercetin to be the main component of SDSZT for the treatment of dry AMD, followed by C2 kaempferol, with a degree of 54, a betweenness of 0.05453, and a closeness of 0.4270, and NZ6 luteolin, with a degree of 32, a betweenness of 0.0705, and a closeness of 0.4318; C1 *β*-sitosterol has a degree of 19, a betweenness of 0.0075, and a closeness of 0.38; SS3*β*-carotene has a degree of 17, a betweenness of 0.0259, and a closeness of 0.3897; B1 stigmasterol had a degree of 14, a betweenness of 0.0214, and a closeness of 0.3535 (Table 3).

PTGS2, with a network degree of 29, a betweenness of 0.1304, and a closeness of 0.5084 in the network, was predicted to be the most important target of SDSZT for the treatment of dry AMD. Next, AKT1, PTGS1, TNF, JUN, BCL2, CASP3, PIK3CG, AR, and BAX were also relatively important regulatory targets (Table 4).

## 4. Discussion

Age-related macular degeneration is an age-related disease that is currently recognized as a high-risk blinding eye disease, and it is essential to establish an effective treatment measure in an increasingly aging society. Dry AMD is known to be a complex disease with multiple risk factors and molecular mechanisms. In the study of in vivo and in vitro experimental models of AMD, these molecular mechanisms involving AMD can be broadly categorized as oxidative stress-mediated, antioxidant dysregulation, inflammation, dysregulation of lipid metabolism, and angiogenic dysregulation [1, 3, 26]. For dry age-related macular degeneration (AMD) and advanced geographic atrophy (GA), the treatment options available only slow the progression of the disease. However, there is currently no therapy that can restore degenerated retinal pigment epithelial (RPE) and/or photoreceptor cells. Currently, studies have focused on antibody, gene, and stem cell therapies for the treatment of dry AMD [27, 28], which are potentially effective therapies but have not yet been applied in the clinical setting in the absence of long-term validated safety profiles.

Shuang Di Shou Zhen Tablets (formerly known as Ziyin Bushen Tablets) have been used in the treatment of dry AMD for 20 years. In previous clinical studies, patients with dry AMD were found to have improved visual acuity after receiving a combination of SDSZT and supplements such as vitamin E and vitamin C compared to before treatment. As the number of deformation grid in Amsler table was reduced, the range of central vision defect was reduced, and there were also statistical differences on these respects between treatment group and control group (only treated with vitamin E and vitamin C) (*P* < 0.05) [6, 7], but the pharmacological mechanism of action has not been clearly understood. In this study, the active components of SDSZT for the treatment of dry AMD were quercetin, kaempferol, luteolin, *β*-sitosterol, *β*-carotene, stigmasterol, etc., based on the preliminary screening by network pharmacology method. Studies on the therapeutic mechanisms of dry AMD have shown that polyphenols such as quercetin can directly scavenge reactive oxygen species (ROS) [29] and it protects ARPE-19 cells against oxidative stress induced by 4-hydroxynonene (HNE), an end product of lipid peroxidation [30]. Quercetin protects RPE cells by decreasing mRNA expression of the proinflammatory IL-6 and IL-8 and monocyte chemotactic protein 1 (MCP-1) [31] and activating the Keap1/Nrf2/ARE pathway [32]. In vivo experiments have shown that kaempferol reduces the apoptosis of rat retinal pigment epithelial cells (RPE cells) caused by NaIO3 by enhancing the expression of vascular endothelial growth factor (VEGF) protein [33]. Luteolin attenuates the IL-1 *β*-induced increase in interleukin 6 (IL-6), interleukin 8 (IL-8), soluble intercellular adhesion molecule 1 (sICAM-1), and monocyte chemotactic protein 1 (MCP-1) production by inhibiting nuclear transcription factor-kB (NF*κ*-B) and MAPK signaling pathways to further protect ARPE19 cells [34]. Therefore, SDSZT may be used to regulate dry age-related macular degeneration through quercetin, kaempferol, and luteolin.

Based on the results of this study, the highest degree value target of SDSZT for dry age-related macular degeneration is PTSG2, while AKT1, PTGS1, TNF, JUN, BCL2, CASP3, PIK3CG, AR, and BAX are also likely to be key therapeutic targets for SDSZT. Gene Ontology analysis and KEGG pathway enrichment analysis showed that these key targets involve multiple pathways, including prostate cancer [35], TNF signaling pathway [36], NF-Kappa B signaling pathway [37], and JAK-STAT signaling pathway [38], all of which are closely related to the AMD development process. *PTGS2*, also known as cyclooxygenase (*COX2*), has been reported to be involved in the AMD process as a key enzyme in the inflammatory response, and is highly expressed in retinal pigment epithelial cells [39]. *JUN*, also known as transcription factor ap-1, can be activated by 4-hydroxyhexenal- (HHE-) under the control of cyanidin-3-glucoside (C3G) and is associated with AMD inflammation [40]. The expression of phosphatidylinositol 3 kinase (*PIK3CG*) was upregulated when bevacizumab treated RPE cells in an AMD model [41], suggesting that *PIK3CG* may be a target for the treatment of AMD. Current information on the modes of RPE death in AMD disease includes apoptosis, necrosis, autophagy, and ferroptosis [42]. The downregulation of the apoptosis suppressor gene *Bcl-2* and the upregulation of the proapoptosis gene *Bax* have been shown to be the main causes of apoptosis in RPE cells in the AMD model [43].

The results of this study indicate that a single monomer compound of SDSZT can regulate different targets, and a single target can interfere with different biological processes and signaling pathways. This embodies the function characteristics of the multipathway, multitarget function of SDSZT. At the same time, it is clear that the whole network can be modulated by modulating a single monomer or an important target or multiple targets in the pharmacological network. This provides a scientific basis for the clinical use of SDSZT for dry age-related macular degeneration and a new direction for exploring the potential mechanisms of SDSZT for dry age-related macular degeneration. However, network pharmacology methods have certain limitations, and validation of the predicted targets and monomer compounds is necessary as well to further demonstrate the clinical therapeutic potential of SDSZT and then clarify the exact regulation target of SDSZT.

## Figures and Tables

**Table 1:** The main ingredients of SDSZT.

Herb	Mol ID	Label	Active ingredient	OB	DL
*Rehmanniae Radix Praeparata*	MOL000359	A1	Sitosterol	36.91	0.75
	MOL000449	B1	Stigmasterol	43.83	0.76

*Mori Fructus*	MOL006209	SS1	Cyanin	47.42	0.99
	MOL000737	SS2	Morin	46.23	−0.77
	MOL002773	SS3	Beta-carotene	37.18	1.52
	MOL000098	D1	Quercetin	46.43	−0.77

*Schisandra chinensis*	MOL009199	WWZ1	Interiotherin B	31.76	0.43
	MOL009213	WWZ5	Kadsulignan B	30.63	0.24
	MOL009219	WWZ8	Neokadsuranic acid C	35.4	0.26
	MOL009220	WWZ9	Kadsulignan	33.35	0.89
	MOL009235	WWZ13	Angusifolin B	34.82	0.42

*Radix Polygoni Multiflori Praeparata*	MOL001771	SW1	Poriferast-5-en-3beta-ol	36.91	0.75
	MOL002268	SW2	Rhein	47.07	0.28
	MOL004373	SW3	Anhydroicaritin	45.41	0.44
	MOL000359	A1	Sitosterol	36.91	0.75

*Paeonia albiflora*	MOL000359	A1	Sitosterol	36.91	0.75
	MOL000358	C1	Beta-sitosterol	36.91	0.75
	MOL000422	C2	Kaempferol	41.88	0.24
	MOL001918	BS1	Paeoniflorgenone	87.59	0.37
	MOL001919	BS2	(3S,5 R,8R,9 R,10S,14S)-3,17-dihydroxy-4,4,8,10,14-pentamethyl-2,3,5,6,7,9-hexahydro-1h-cyclopenta[a]p	43.56	0.53
	MOL001924	BS3	Paeoniflorin	53.87	0.79
	MOL000211	BS4	Mairin	55.38	0.78
	MOL000492	BS5	(+)-catechin	54.83	0.24

*Rhizoma Dioscoreae*	MOL000449	B1	Stigmasterol	43.83	0.76
	MOL001559	SY1	Piperlonguminine	30.71	0.18
	MOL001736	SY2	(-)-Taxifolin	60.51	0.27
	MOL000322	SY3	Kadsurenone	54.72	0.38
	MOL005430	SY4	Hancinone C	59.05	0.39
	MOL005435	SY5	24-Methylcholest-5-enyl-3belta-O-glucopyranoside_qt	37.58	0.72
	MOL005438	SY6	Campesterol	37.58	0.7
	MOL005440	SY7	Isofucosterol	43.78	0.76
	MOL005458	SY8	Dioscoreside C_qt	36.38	0.87
	MOL000546	SY9	Diosgenin	80.88	0.81
	MOL005465	SY10	AIDS180907	45.33	0.77
	MOL000953	SY11	CLR	37.87	0.68

*Alisma orientale*	MOL000359	A1	Sitosterol	36.91	0.75
	MOL000831	ZX1	Alisol B monoacetate	35.58	0.81
	MOL000849	ZX2	16*β*-methoxyalisol B monoacetate	32.43	0.77
	MOL000853	ZX3	Alisol B	36.76	0.82
	MOL000856	ZX4	Alisol C monoacetate	33.06	0.83
	MOL002464	ZX5	1-Monolinolein	37.18	0.3
	MOL000862	ZX6	[(1S,3 R)-1-[(2R)-3,3-dimethyloxiran-2-yl]-3-[(5R,8S,9S,10S,11S,14 R)-11-hydroxy-4,4,8,10,14-pentamethyl-3-oxo-1,2,5,6,7,9,11,12,15,16-decahydrocyclopenta[a]phenanthren-17-yl]butyl] acetate	35.58	0.81

*Ligustrum lucidum*	MOL000358	C1	Beta-sitosterol	36.91	0.75
	MOL000422	C2	Kaempferol	41.88	0.24
	MOL000098	D1	Quercetin	46.43	−0.77
	MOL004576	NZ1	Taxifolin	57.84	0.27
	MOL005146	NZ2	Lucidumoside D	48.87	0.71
	MOL005147	NZ3	Lucidumoside D_qt	54.41	0.47
	MOL005190	NZ4	Eriodictyol	71.79	0.24
	MOL005212	NZ5	Olitoriside_qt	103.23	0.78
	MOL000006	NZ6	Luteolin	36.16	0.25

*Ophiopogon japonicus*	MOL001659	MD1	Poriferasterol	43.83	0.76
	MOL000631	MD2	Coumaroyltyramine	112.9	0.2

*Radix Rehmanniae*	MOL002813	DH1	Aucubin	35.56	0.33
	MOL012254	DH2	Campesterol	37.58	0.71

**Table 2:** Enrichment analysis of candidate targets for SDSZT against dry AMD.

GO	Description	Count	Log10(P)	Hits
hsa05200	Pathways in cancer	52	−58.12	AKT1|AKR1B1|AR|BAX|CCND1|BCL2|CASP3|CASP7|CASP8|CDK2|CDKN1A|CTNNB1|NQO1|E2F1|E2F2|EDN1|EGF|EGFR|ERBB2|ESR1|F2|FOS|GSTM1|GSTP1|HIF1A|HMOX1|HSP90AA1|IFNG|IGF2|IL2|IL4|IL6|CXCL8|JUN|MDM2|MMP2|MMP9|MYC|NFE2L2|NOS2|PPARD|MAPK1|MAPK8|PTEN|PTGS2|RAF1|RB1|RELA|STAT1|TGFB1|TP53|VEGFA

ko05418	Fluid shear stress and atherosclerosis	31	−45.9	AKT1|BCL2|CAV1|MAPK14|CTNNB1|NQO1|EDN1|FOS|GSTM1|GSTP1|HMOX1|HSP90AA1|ICAM1|IFNG|IL1A|IL1B|JUN|KDR|MMP2|MMP9|NFE2L2|NOS3|PLAT|MAPK8|RELA|CCL2|SELE|TNF|TP53|VCAM1|VEGFA

hsa05215	Prostate cancer	25	−38.22	AKT1|AKR1B1|AR|CCND1|BCL2|CDK2|CDKN1A|CTNNB1|
				E2F1|E2F2|EGF|EGFR|ERBB2|GSTP1|HSP90AA1|MDM2|MMP3|MMP9|PLAT|MAPK1|PTEN|RAF1|RB1|RELA|TP53

ko04668	TNF signaling pathway	23	−33.56	AKT1|CASP3|CASP7|CASP8|MAPK14|EDN1|FOS|CXCL2|ICAM1|IL1B|IL6|CXCL10|JUN|MMP3|MMP9|MAPK1|MAPK8|PTGS2|RELA|CCL2|SELE|TNF|VCAM1

hsa05205	Proteoglycans in cancer	24	−27.71	AKT1|CCND1|CASP3|CAV1|CDKN1A|MAPK14|CTNNB1|EGFR|ERBB2|ESR1|HIF1A|IGF2|IL6|KDR|MDM2|MMP2|MMP9|MYC|MAPK1|RAF1|TGFB1|TNF|TP53|VEGFA

hsa05210	Colorectal cancer	17	−23.46	AKT1|BAX|CCND1|BCL2|CASP3|CDKN1A|CTNNB1|EGF|EGFR|FOS|JUN|MYC|MAPK1|MAPK8|RAF1|TGFB1|TP53

hsa04932	Nonalcoholic fatty liver disease	18	−20.48	AKT1|BAX|CASP3|CASP7|CASP8|IL1A|IL1B|IL6|CXCL8|INSR|JUN|PPARA|MAPK8|PTGS1|PTGS2|RELA|TGFB1|TNF

ko05144	Malaria	13	−20.39	CD36|CD40LG|ICAM1|IFNG|IL1B|IL6|CXCL8|IL10|CCL2|SELE|TGFB1|TNF|VCAM1

hsa01524	Platinum drug resistance	13	−17.9	AKT1|BAX|BCL2|CASP3|CASP8|CDKN1A|ABCC2|ERBB2|GSTM1|GSTP1|MDM2|MAPK1|TP53

hsa04630	JAK-STAT signaling pathway	14	−14.36	AKT1|CCND1|BCL2|CDKN1A|EGF|EGFR|IFNG|IL2|IL4|IL6|IL10|MYC|RAF1|STAT1

ko04064	NF-kappa B signaling pathway	11	−13.05	PARP1|BCL2|CD40LG|CXCL2|ICAM1|IL1B|CXCL8|PTGS2|RELA|TNF|VCAM1

hsa05202	Transcriptional misregulation in cancer	13	−11.96	BAX|CDKN1A|IGFBP3|IL6|CXCL8|MDM2|MMP3|MMP9|MPO|MYC|PLAT|RELA|TP53

hsa04931	Insulin resistance	10	−10.71	AKT1|CD36|IL6|INSR|NOS3|PPARA|MAPK8|PTEN|RELA|TNF

hsa04217	Necroptosis	11	−10.31	PARP1|BAX|BCL2|CASP8|HSP90AA1|IFNG|IL1A|IL1B|MAPK8|STAT1|TNF

hsa05216	Thyroid cancer	7	−9.87	BAX|CCND1|CDKN1A|CTNNB1|MYC|MAPK1|TP53

hsa04371	Apelin signaling pathway	10	−9.6	AKT1|CCND1|NOS2|NOS3|SERPINE1|PIK3CG|PLAT|MAPK1|RAF1|SPP1

hsa04062	Chemokine signaling pathway	11	−9.45	AKT1|CXCL2|CXCL8|CXCL10|PIK3CG|MAPK1|RAF1|RELA|CCL2|CXCL11|STAT1

ko05330	Allograft rejection	6	−8.1	CD40LG|IFNG|IL2|IL4|IL10|TNF

ko05120	Epithelial cell signaling in *Helicobacter pylori* infection	7	−8.11	CASP3|MAPK14|EGFR|CXCL8|JUN|MAPK8|RELA

ko00380	Tryptophan metabolism	6	−8.03	CAT|CYP1A1|CYP1A2|CYP1B1|MAOA|MAOB

**Table 3:** SDSZT main active ingredient network node characteristic. The chemical structure diagram is from the TCMSP Database.

MOLID	Molecule name	Degree	Betweenness	Closeness	Chemical structure
MOL000098	Quercetin	162	0.4159	0.608	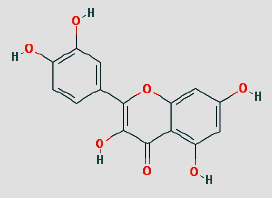
MOL000422	Kaempferol	54	0.05453	0.4270	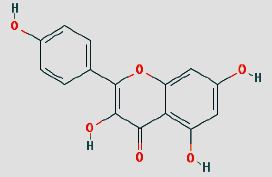
MOL000006	Luteolin	32	0.0705	0.4318	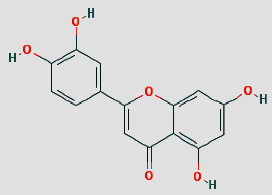
MOL000358	Beta-sitosterol	19	0.0075	0.38	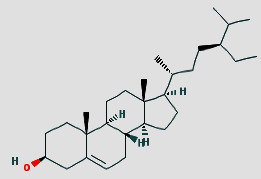
MOL002773	Beta-carotene	17	0.0259	0.3897	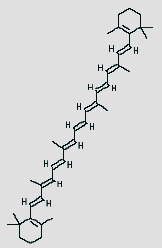
MOL000449	Stigmasterol	14	0.0214	0.3535	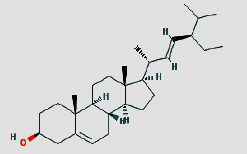

**Table 4:** SDSZT main active component target network node characterization parameters.

Target	Degree	Betweenness	Closeness
PTGS2	29	0.13042	0.50836
AKT1	19	0.02642	0.46200
PTGS1	19	0.05248	0.47059
TNF	15	0.02098	0.44058
JUN	15	0.01317	0.44060
BCL2	15	0.01198	0.43804
CASP3	15	0.01202	0.44315
PIK3CG	13	0.01881	0.43304
AR	13	0.03148	0.44838
BAX	13	0.00913	0.42577

## Data Availability

The data supporting this network pharmacology analysis are from previously reported studies and databases, which have been cited. The processed data are available from the corresponding author upon request.
